# 

**Published:** 1809-05

**Authors:** 


### NEW MEDICAL PUBLICATIONS.

An Inquiry into the Laws of Epidemics; with Remarks on the Plans lately proposed for exterminating the Small-pox. By Joseph Adams, M. D. F. L. S. 5s. 6d.

A Short Treatise on the Virtues of Dr. Gordon's Vegetable Balsamic Pills. 1s.

Suggestions for the Prevention of the Yellow Fever. To which is added, the Outline of a Plan of Military Hospitals, tending to a more successful Treatment of the Sick. By Stewart Henderson, M. D. District Staff Surgeon. Royal 8vo. 5s.

A Dictionary of Practical Surgery. By Thomas Cooper, Member of the Royal College of Surgeons in London. 8vo. 15s.

A Practical Materia Medica, in which the various Articles are fully described, and divided into Classes and Orders, according to their Effects. 12mo. 5s.

